# Causal Relationship between Diet-Induced Gut Microbiota Changes and Diabetes: A Novel Strategy to Transplant *Faecalibacterium prausnitzii* in Preventing Diabetes

**DOI:** 10.3390/ijms19123720

**Published:** 2018-11-22

**Authors:** Kumar Ganesan, Sookja Kim Chung, Jairam Vanamala, Baojun Xu

**Affiliations:** 1 Food Science and Technology Program, Beijing Normal University—Hong Kong Baptist University United International College, Zhuhai 519087, China; kumarg@hku.hk (K.G.); skchung@uic.edu.hk (S.K.C.); 2 School of Biomedical Sciences, The University of Hong Kong, Hong Kong, China; 3 Department of Food Science, Pennsylvania State University, University Park, State College, PA 16801, USA; jairam.vanamala@gmail.com

**Keywords:** diet, gut microbiota, diabetes, *Faecalibacterium prausnitzii*, novel strategies

## Abstract

The incidence of metabolic disorders, including diabetes, has elevated exponentially during the last decades and enhanced the risk of a variety of complications, such as diabetes and cardiovascular diseases. In the present review, we have highlighted the new insights on the complex relationships between diet-induced modulation of gut microbiota and metabolic disorders, including diabetes. Literature from various library databases and electronic searches (ScienceDirect, PubMed, and Google Scholar) were randomly collected. There exists a complex relationship between diet and gut microbiota, which alters the energy balance, health impacts, and autoimmunity, further causes inflammation and metabolic dysfunction, including diabetes. *Faecalibacterium prausnitzii* is a butyrate-producing bacterium, which plays a vital role in diabetes. Transplantation of *F. prausnitzii* has been used as an intervention strategy to treat dysbiosis of the gut’s microbial community that is linked to the inflammation, which precedes autoimmune disease and diabetes. The review focuses on literature that highlights the benefits of the microbiota especially, the abundant of *F. prausnitzii* in protecting the gut microbiota pattern and its therapeutic potential against inflammation and diabetes.

## 1. Introduction

Microorganisms inhabit many human body sites mostly residing in the GI tract (Gastrointestinal tract), which confers metabolic, immunological and neurological advantages [1]. Humans are now said to be ‘superorganisms’, based on the residential microbial genetic material (microbiome) accompanying the human genome. It is known that our microbiota develops and alters human gene expression in response to adapting to new environmental settings [2]. Moreover, the gut microbes contribute to efficient energy metabolism, which confers selective benefits during starvation [3]. It has been proposed that human gut comprises in the range of 1000 bacterial species with different phyla. The bacterial species are mainly members of the phyla *Firmicutes* and *Bacteroidetes* being also present, although at lower amounts, other phyla such as *Actinobacteria*, *Proteobacteria* and *Verucomicrobia* [4]. *Firmicutes* constitute the largest percentage (60%), with almost 200 genera, composed of *Lactobacillus*, *Mycoplasma*, *Bacillus*, *Eubacterium*, *Faecalibacterium*, *Ruminococcus*, *Roseburia*, and *Clostridium*. *Firmicutes* are recognized as the predominant producers of butyrate in the gut and special degraders of indigestible polysaccharides [5]. *Bacteroidetes* are in smaller proportions (10%) (includes *Alistipes*, *Bacteroides*, *Parabacteroides*, *Porphyromonas*, *Prevotella*), which utilize a huge quantity of substrates and are primary producers of propionate [6,7]. *Actinobacteria* (*Bifidobacterium* and *Collinsella*), *Betaproteobacteria* (*Escherichia coli* and *Desulfovibrio*), *Verrucomicrobia* (*Akkermansia muciniphila*) and *Fusobacteria* are also typically present in smaller numbers in the healthy gut [7]. Overall, these bacterial communities play a vital role to facilitate a healthy gut microbiota pattern.

**Figure 1:**
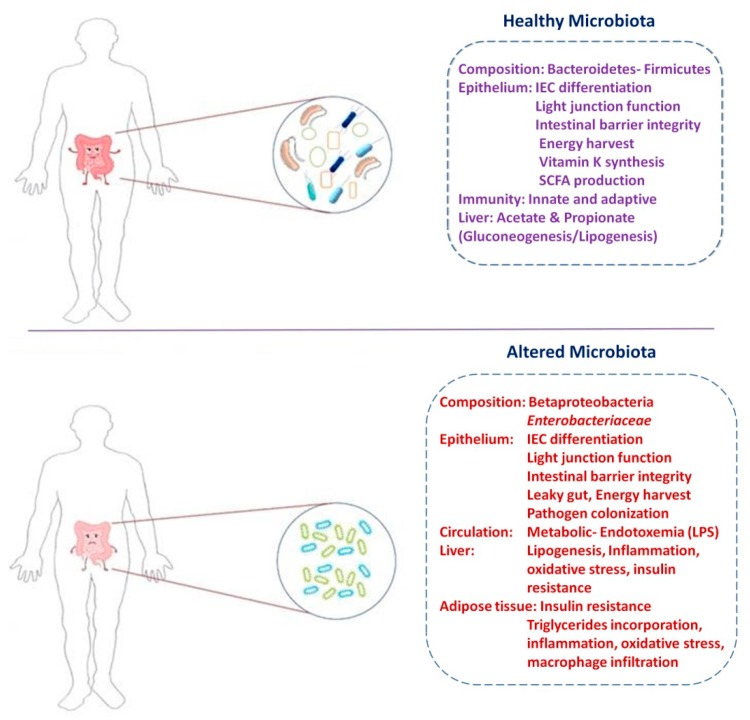
Healthy gut microbiota versus the altered microbiota. Based on Patterson et al [34], healthy gut microbiota composed of predominant phyla Firmicutes (60%) to Bacteroidetes, which restricts lipopolysaccharide (LPS) translocation by the integrity of the intestinal epithelial barrier and harvest energy for the host. Unhealthy microbiota profile causes metabolic dysfunction in peripheral organs, leading to increased adiposity, chronic inflammation, oxidative stress, diabetes, and obesity. In addition, the secretion of gut hormones (incretins ghrelin, amylin) can affect metabolic syndrome and diabetes [19,34,35]. IEC, intestinal epithelial cell; GLP-1, glucagon-like peptide-1; GIP, gastric inhibitory peptide; SCFA, short chain fatty acid.

However, the healthy gut ecosystem could be altered due to an alteration of microbial compositions, which are largely due to the dietary patterns (vegetarian and Western), antibiotics, probiotics, and lifestyle [7,8]. During early development to adult, the changes in the dietary compositions of high-fat diet through the intake of mother’s milk in newborns to the introduction of carbohydrate-rich solid and complex diet reestablish and stabilize the microbiotic community similar to that of an adult. Microbiota in adults is also relatively stable until the persons get 60 years old [8]. About 30% of the microbial communities are represented as cultured isolates, and the remaining is probably capable of being cultured [9]. Alterations of these microbial communities are extremely connecting with various diseases. These alterations lead to elevated gut permeability and reduced gut mucosal immunity, contributing to the development of various cancers [10,11,12], autoimmune disorders [13,14,15], inflammatory bowel diseases [16,17,18], metabolic syndrome [19,20,21,22,23,24,25,26,27,28] and neurodegenerative diseases [29,30,31,32,33]. In addition, the elevated intestinal permeability is consequences of reduced expression of tight junction proteins that may favor to the uncontrolled passage of antigens. It enables the translocation of bacterial lipopolysaccharide to the gut connective tissues and to the blood circulation, which can cause insulin resistance and metabolic endotoxemia [34] (Figure 1).

There is an intricate relation between dietary nutrients and the bacterial communities. Gut microbiota co-evolved with host organisms to provide unique metabolic functions, as reflected in broad patterns of food consumption and energy-yielding through distinct microbes [36]. Diet plays a main role in shaping gut microbiota through the delivery of energy and contributes to microbial growth [37,38]. Microbiota is able to break down polysaccharides that are non-digestible by humans and provides a wide range of metabolites (including SCFAs), which help to maintain the gut ecosystem [39,40]. Therefore, the diet has a greater function to manage a number of clinical manifestations through the microbiota [41]. Recent studies showed that the diet (low protein and carbohydrates) involves not only maintaining healthier gut ecosystems, but also stabilizing the microbiota, gut mucosal immunity and effective for insulin resistance therapies [35,42].

## 2. Interactions between the Gut Microbiota and Dietary Nutrients

**Figure 2:**
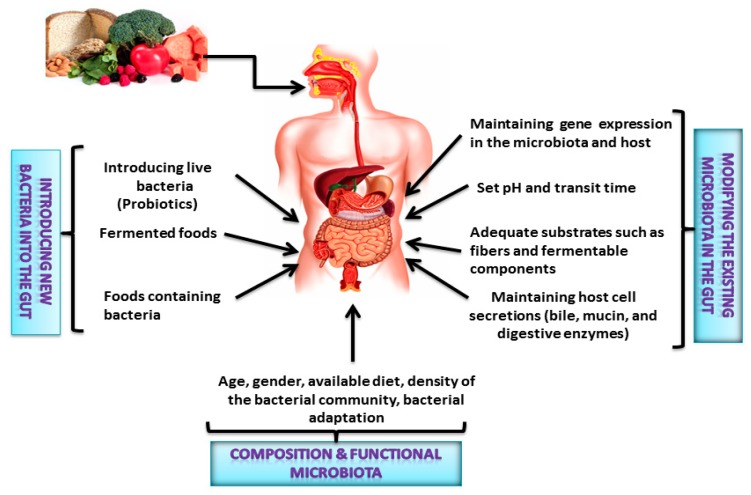
Dietary patterns, diet composition, and probiotics determine colonic microbiota composition and functions.

Diet is considered as the primary modulators of the human gut microbiota. Plant-derived complex carbohydrates, such as the resistant starch, beta-glucans, heteropolysaccahrides, and dietary fibers from cereals, legumes, vegetables, fruits, and nuts, cannot be completely digested by the human digestive tract. Gut microorganisms are able to synthesize some exoenzymes to catalyze and ferment the complex polysaccharides from botanical food to facilitate digestion in a host gut to produce adequate quantities of bacterial metabolites (SCFAs) [43]. These SCFAs generally have a beneficial effect on the gut and systemic health of the host [44]. For instance, *Bifidobacterium* helps to prevent pathogenic infection through the production of acetate [45], and *Faecalibacterium prausnitzii*, an important butyrate-producer, protects from inflammation in the gut [46]. However, several animal studies have demonstrated that dietary changes provide tremendous alterations in the compositions of the microbiota that leads to various illnesses [44,47,48,49]. Hence, these gut bacteria appear pivotal in mediating the health effects of foods. Various mechanisms are proposed for the impact of food associated with colonic microbiota composition and functions (Figure 2).

The diet compositions regulate several biochemical factors whose primary functions keep a role in the modulation of the microbiota. For instance, fiber diet consumption not only elevates a number of fermentable substrates in the host, but also diminishes the luminal pH and enhances the transit rate with excess acid production. Based on the accelerated transit and acidic environment, *Bacteroides* are growing rapidly [50]. The gastrointestinal tract pH normally ranges between 5 and 5.5 in the ileum and the colon has a range from 6.6 to 7.0, which is one of the main factors in constructing the shape of the microbial communities in the colon. Diet compositions containing fermentable polysaccharides are regulators of the intestinal pH, which facilitates a more acidic environment through the end-products of SCFAs in the gut [44]. Zimmer et al. [51] have found that the pH of the stool from vegetarian diets (144 subjects) mean values of 6.3 and the omnivores have the mean pH of 6.8 (105 subjects). This study showed an increase in the bacterial count of *Bacteroides* and *Bifidobacterium* in vegetarian diet consumers compared with omnivore’s individuals. However, the pH ranges (≤6.3) do not support bacteria, such as *E. coli* and *Enterobacteriaceae* in their growth as they prefer pH ranges >6.5 [44]. Hence, dietary habit and the increased fiber intake cause lower pH through augmented bacterial metabolites in vegetarians, which may be directly responsible for lower counts of these bacteria. Furthermore, these oragniams prefer proteins as the primary source of energy that explains their higher counts in omnivores [51]. The stool pH becomes more alkaline, with the increase in age and differs significantly between genders [29,52]. Higher consumption of animal protein is one possible mechanism for higher stool pH in subjects on omnivores. This alkalinity is generally caused due to its alkaline metabolites produced by proteolytic putrefactive bacteria, such as *Bacteroides*, *Propionibacterium*, *Streptococcus*, *Clostridium*, *Bacillus*, and *Staphylococcus* [53]. In addition to age, gender, and nutrients, and factors including microbial interaction, food passage through different intestinal compartments with diverse bacterial colonization mass, sulfate, bile acids, and bacterial adaptation, may all affect the conformation and activity of the colonic microbiota [51].

Bile is another essential factor that indirectly impacts digestions. Bile acids are cholesterol derived detergents, play the main role in the digestion and absorption of fats, lipid transporter, and turnover, as well as detoxification. They are antibacterial and create strong selective forces on the gut microbiota, even within a single species, exhibit differential sensitivity [54]. The fat and protein contents of the diet regulate the excretion of bile and can thus indirectly shape the microbiota [44]. Mucins secreted from goblet cells and digestive enzymes of pancreatic origin represent substantial polysaccharide and protein sources for the gut microbes and assist in the normal turnover of the mucus barrier lining the gut [55]. Several *Bacteroides*, *Bifidobacteria*, and *Akkermansia muciniphila* can degrade the mucin, provide a more stable resource that may contribute half of the carbon flux in the intestinal tract [56]. Beneficial bacteria that we eat in food (probiotics) can also contribute to the luminal microbiota. In infants, the breast milk-derived bacteria readily colonize the gut [57]. In adults, the well-organized microbiota possesses high colonization resistance and low susceptibility to non-indigenous species. The fermented and probiotic supplements are believed to confer their health benefits in the host and involve modification of the indigenous microbiota functional activity [58]. Most probiotic studies show that their health benefits and the ability to re-shape the microbiota are unclear [54,55,56,57,58].

The impact of diet on host gene expression and its possible effects on the microbiota have been summarized by Luo et al. [59]. The impacts of carbohydrate diet on the gene expression have focused on *Bacteroidetes* [57] and protein diets with *Escherichia*/*Shigella*, *Enterococcus*, *Streptococcus*, and sulfate-reducing bacteria [60]. In vivo transcriptional profiling study has also confirmed that the substrate-specific, glycan-metabolizing genes are expressed upon inducible manner. Gut microbiota community plays to be very stable and more influenced by dietary sources than by genetic factors [44]. The consumption of high-fat plus high-protein diet increases the abundance of *Bacteroidetes* and *Prevotella* [61,62]. However, dietary intervention has also altered the microbiota composition [63]. In fact, a high-fat plus high-carbohydrate meal induces comprehensive endotoxemia and inflammation in the gut [64]. However, the consumption of high-fruit plus high fiber meal or orange juice or a polyphenol preparation with resveratrol does not cause any side-effects, including endotoxemia and inflammation [65,66].

The composition of microbial communities differs greatly among individuals. An individual generally represents a unique collection of genera and sub-species and it may be different based on the diet (vegetarian or Western with high protein or fat), the age of the host organism, genetic and environmental factors [67]. Diet provides nutrients for not only the host, but also provides energy to the microbial community. Hence, the diet greatly influences the diversity of the microbiota in the gut (Table 1). The microbiota is genetically well equipped to utilize various nutritional substrates [68] and maintains the normal gut microbiota pattern. A recent study has also shown that an increase in fat consumption generates a more gram-positive/gram-negative index of the gut microbiota [8]. These microbiota numbers would be double within an hour based on the available nutrients [47].

**Figure 3:**
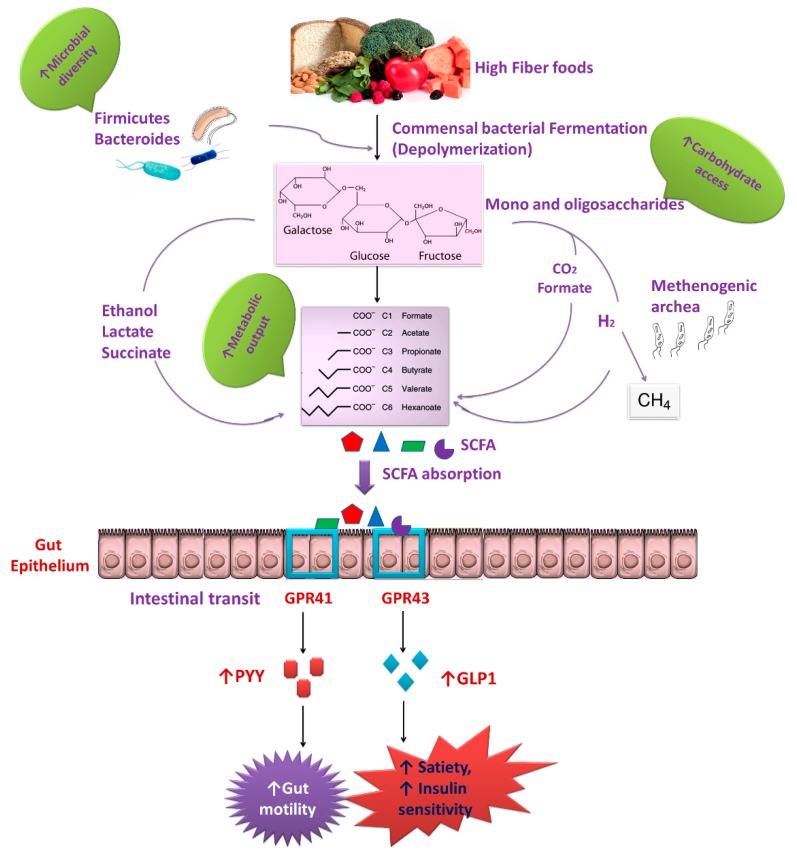
Dietary fiber is a source of complex carbohydrates, which are required for the production of SCFA. When the diversity of the microbiota is high, the accessible rate of complex carbohydrates is relatively high. The production of multiple types of SCFA helps not only energy source for a host and microbiota, but also to recruit additional diversity to the gut microbiota. SCFA is also a substrate for gluconeogenesis, which modulates central metabolism, and are involved in signaling to the host by activating G-protein-coupled receptors, such as GPR41 and GPR43, which triggers the release of the hormone GLP1secretion, increasing insulin sensitivity, and inducing satiety [141]. On the other hand, GPR41 activate peptide YY (PYY), an intestinal hormone that influences gut motility, enhances intestinal transit rate, and decreases energy harvest from the diet [139]. Butyrate can elevate the regulatory T cells (Tregs), thus suppress chronic inflammation.

Complex diet enhances the production of various types of SCFAs and adds diversity to the gut microbiota. SCFAs production is normally associated with the greater number of *Bacteroides* species, which is a consistent producer of propionate [128]. The propionate possesses potent health-promoting effects, which includes anti-lipidemia, anti-inflammatory, immunomodulatory, and anti-cancer activities [129]. The fiber-containing nutrients have been reported to reduce colon pH and to enhance the diversity of the microbiota [130]. Microbial population metabolizes dietary fiber into oligosaccharides, which are further fermented into SCFAs, such as butyrate, acetate, and propionate, which activate the G-protein-coupled receptors (GPCR), GPR41 presents in the gut and GPR43 is only expressed by the epithelial cells. Interestingly, the phenotypes of mice with the deletion of GPR41 and GPR43 presented altered chronic inflammation and obesity markers, which suggested that these GPCRs are important regulators of chronic inflammation in the gut, respiratory tract and skeletal system and metabolic dysregulation leading to obesity [131]. Binding of ligands to GPR41 may trigger secretion of glucagon-like peptide 1 (GLP-1) and lead to improve insulin sensitivity and satiety (Figure 3). GLP-1 secretion stimulated by GPR43 is dependent on the presence of nutrients in the lumen and microbial communities in the gut.

GLP-1 upon binding to its receptor on pancreatic β-cells can increase the cAMP level and activate protein kinase A (PKA) or cAMP-regulated guanine nucleotide exchange protein activated by cAMP (Epac1 and Epac2), which in turn activates insulin secretion by stimulating Ca^2+^ signaling [132]. Between two isoforms of Epac, it was believed that Epac2 is more abundantly expressed in β-cells of the pancreas; however, our and other studies have reported that Epac1 is also expressed by the β-cells [133,134]. The expression of both isoforms, Epac1 and Epac2 are elevated after exogenous treatment of exendin-4 (Ex-4), a dipeptidyl peptidase IV (DPP-IV)-resistant GLP-1 analog, which promotes differentiation of fetal pancreatic tissue, pancreatic progenitors, and intestinal stem cells into insulin-producing cells and ameliorates hyperglycemia [135,136]. In addition, exogenous treatment of Ex-4 also leads to increased insulin secretion in β-cells differentiated from mouse embryonic stem cells, with increased expression of insulin-1, pancreatic and duodenal homeobox 1 (PDX-1), sulfonylurea receptor 1 (SUR1; a subunit of the ATP-sensitive K^+^ (K_ATP_) channel), Epac1, and Epac2 [137]. The critical experiment to show the importance of Epac 1 in mediating the GLP-1 signal and metabolic syndrome and diabetes was performed using the genetically engineered Epac1-deficient mice and embryonic stem (ES) cells [133]. The homozygous Epac1-knockout (*Epac1*^−/−^) mice, which are slightly heavier, developed impaired glucose tolerance and GSIS and less insulin sensitivity with altered islet cytoarchitecture of pancreatic islets. After the high-fat diet, these Epac1 deficient mice become more obviously heavier and significantly higher in GSIS. Moreover, *Epac1*^−/−^ mice developed severe hyperglycemia with increased β-cell apoptosis and insulitis after type1 and immune model of diabetes using the multiple low-dose streptozotocin (MLDS; 40 mg/kg) treatment than *Epac1*^+/+^ mice. Interestingly, *Epac1*^−/−^ mice also showed metabolic syndrome, with an increased respiratory exchange ratio and plasma triglyceride, and more severe diet-induced obesity with insulin resistance, which may contribute to *β*-cell dysfunction and insulin secretion. Nevertheless, islets distinguished from *Epac1*^−/−^ ES cells exhibited insulin secretion flaw, decreased Glut2 and PDX-1 expression, and eliminated GLP-1-stimulated PCNA induction, signifying a numerous role of Epac1 in β-cell function. Although the investigations provided in-vitro and in vivo evidence that Epac1 has a key role in glucose homeostasis and β-cell function, it is not clear whether these Epac1 deficient mice have any defect in the GLP-1 secretion by the L-cells in the gut after a meal [138]. Therefore, the GLP-1 signal through Epac1 in the gut and their role as a potent antihyperglycemic hormone; secretagogues the β-cells of the pancreas secrete insulin, which lowers the blood glucose. In addition, GLP-1 inhibits glucagon secretion in α-cells of the pancreas [139]. On the other hand, GPR41 activates peptide YY (PYY), an intestinal hormone that influences gut motility, enhances intestinal transit rate, and decreases energy harvest from the diet [130]. In addition, Epac1 role in cell–cell interaction and junction [140] may help to maintain the epithelial tight and adhesion junctions thereby preventing leaky epithelial lining of the gut. Such condition may also alter the gut microbiota. Currently, this hypothesis is being investigated in our laboratory using the Epac1, Epac2 or double mutant mice and comparing the changes to wild-type mice.

## 3. Role of Gut Microbiota in Diabetes

Gut microbiota compositions are connected with various hallmarks of metabolic dysfunctions, including obesity, and type-2 diabetes. Studies suggest that gut microbes contribute to the onset of the low-grade inflammation characterizing these metabolic disorders via mechanisms associated with gut barrier dysfunctions [142]. The gut barrier generally regulates the permeability of the intestinal mucosa. Disruption of the gut barrier gives rise to enhanced gut permeability and causes leaky gut [143]. The intestinal mucosa is a primary site for pathogen invasion since, when undamaged, it provides the first line of defense against microbial pathogens [144]. Increased intestinal mucosa permeability and loss of integrity may facilitate enteric bacterial pathogens that contain lipopolysaccharide (LPS) crossing of the bloodstream, which can directly damage pancreatic β-cells [145] and accelerates insulitis in animal models [146,147,148]. It was further observed that the increased gut permeability occurs in all rodent strains based on the age and susceptibility of the infection of the animals [62]. Furthermore, it can allow greater exposure to the immune system of diet or pathogenic antigens, triggering low-grade inflammation and immune-mediated destruction of pancreatic β-cells eventually causes diabetes [28,149,150,151]. Generally, an adequate SCFA (butyrate) production levels are essential for gut integrity [152]. The butyrate-producing bacteria, such as *Eubacterium*, *Fusobacterium*, *Anaerostipes*, *Roseburia*, *Subdoligranulum*, and *Faecalibacterium*, have the potential of anti-inflammatory effect both *in vitro* and *in vivo* investigations [153,154]. These bacterial species help to reduce bacterial translocation, improve the organization of tight junctions and stimulate the secretion of mucin to maintain the integrity of the gut, with beneficial effects against inflammation in the gut [155]. However, any alterations in these gut microbiota, in either composition and/or functional, are strongly associated with β-cell autoimmunity and insulin resistance [67] (Table 2).

**Figure 4:**
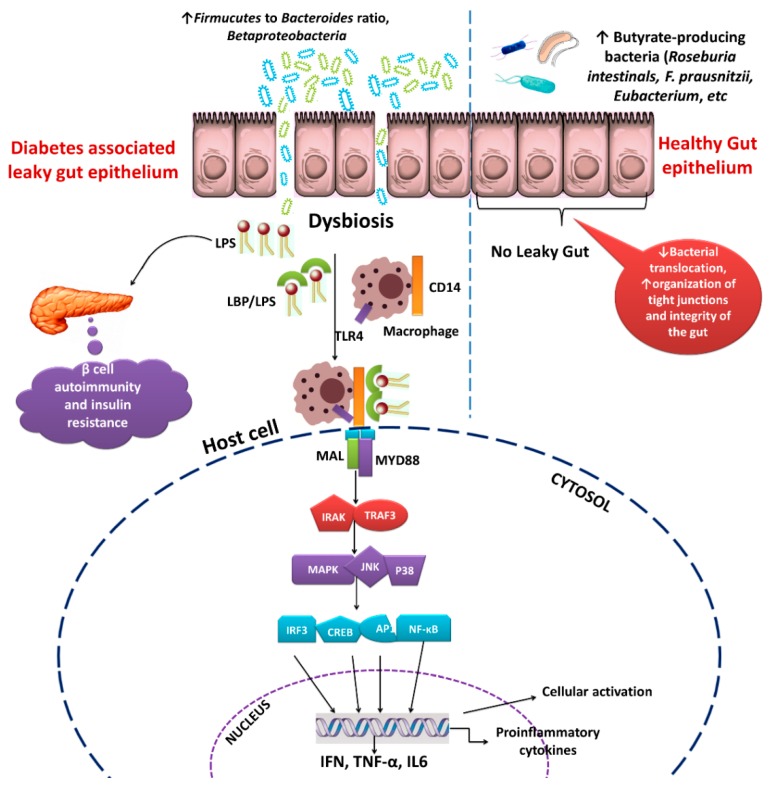
Altered microbial communities enhance the gut permeability and cause leaky gut. The lipopolysaccharide binding protein (LBP), synthesized from the liver, acts as a carrier of LPS. LPS is the primary constituents of the outer membrane of intestinal bacteria, known to cause chronic inflammation in the host. LPS/LPB complex assembles with membrane-bound CD14 (cluster of differentiation 14) molecules and toll-like receptor 4 (TLR4) on the surface of macrophages in the host. TLR4signaling is initiated by ligand-induced dimerization of receptors, which engage with adaptor proteins like MYD88 (myeloid differentiation primary response protein 88) and MAL (MYD88-adaptor-like protein). These downstream signaling pathways stimulate the connections among IL-1R-associated kinases (IRAKs) and the adaptor molecules TNF receptor-associated factors (TRAF). The association of these molecules triggers the mitogen-activated protein kinases (MAPK), JUN N-terminal kinase (JNK) and p38, and subsequently activate the transcription factors, such as nuclear factor-κB (NF-κB), interferon regulatory factors (IRF), cyclic AMP-responsive element-binding protein (CREB) and activator protein 1 (AP1) [168,169]. TLR4 signaling downstream pathways induce pro-inflammatory cytokines that impair insulin secretion and insulin mRNA expression in human beta cell islets [175]. NF-κB could also inhibit insulin gene expression by interacting with CREB [160].

More abundant flora of the class *Betaproteobacteria* was found in the gut of individual with type-2 diabetes as compared to the non-diabetic individual [27]. In animals, the ratios of *Firmicutes* to *Bacteroidetes* were higher in diabetic rats while compared with normal rats [139]. Gut microbiota grows mainly based on dietary nutrients, and its metabolites, which in turn modulate host mucosal immunity through downstream mechanisms, including stimulation of regulatory T-cells and cause pro-inflammatory signals [166]. LPS, a major cell wall component of Gram-negative bacteria, is known to be potent endotoxin in inducing chronic inflammation [167]. When binding to CD14 and Toll-like receptor4 (TLR4) on the surface of macrophage, a high concentration of LPS can initiate a downstream series of inflammatory mechanisms [35,168]. Specific molecular proteins, such as c-Jun N-terminal kinase (JNK) and p38 mitogen-activated protein kinase (MAPK) control the effects of inflammation and insulin signaling [169,170]. Hochdorfer et al. [171] also found that MAPK signaling is important to the development of type-2 diabetes, due to alteration of gut microbiota, which causes leaky gut. In addition, the activation of JNK and p38 can be triggered in diabetic subjects due to oxidative stress in various tissues [172]. The p38 can be generally activated by high glucose concentrations in diabetic subjects [173] and therefore, the levels of JNK and p38 were elevated in the diabetic subjects, indicating that the disturbed gut microbiota is associated with elevated MAPK signaling [174]. Furthermore, inflammation is one of the major pathophysiological factors leading to insulin resistance and progressively causes type-2 diabetes (Figure 4).

## 4. Multi-Skilled *Commensal bacterium F. prausnitzii*: A Diagnostic and Therapeutic Biomarker for Gut-Associated Diseases

*F. prausnitzii* is a multi-skilled commensal organism and a chief member of human microbiota. It is broadly distributed in the digestive tract of mammals and also in some insects. FISH analyses in pigs revealed that *F. prausnitzii*-associated bacteria is exactly similar to that in humans [176]. It is rich in the hind gut rather than in the stomach, as well as jejunum [177]. Generally, *F. prausnitzii* has been found in the guts of chickens and turkeys [178], pigs and piglets [176], calves [179], rats and mice [180]. The assessment of gut microbiota aids to support diagnosis and/or therapeutic tool for various intestinal diseases, which has increased attention during the last few years. Various studies reported that the abundance of fecal or mucosa-related *F. prausnitzii* is a possible biomarker for various gut-associated disorders [182,183,184,210]. In specific, *F. prausnitzii* is a possible biomarker for inflammatory bowel disease, Crohn’s disease, and Colitis (Table 3).

## 5. Dietary Interventions Modulate *F. prausnitzii*

**Figure 5:**
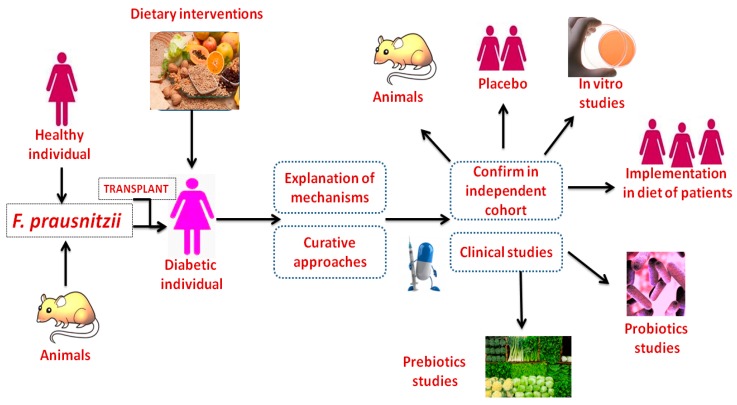
Novel strategies for diabetes prevention by dietary intervention and a transplant of *F. prausnitzii* to the diabetic individual—Isolation of *F. prausnitzii* is either from experimental animals or healthy individual and introduce into diabetic persons through the infusion of the stool or by mouth in the form of a capsule. The initiation step for the identification of a strategy to adapt the gut flora is through components of the diet interventions. Appropriate experimental studies (in vitro, placebo or animal models) and elements in independent cohorts are used to explain the principal mechanisms and to pilot curative approaches to modulating the intestinal bacteria, which laid the foundations for probiotics or prebiotics trials in humans to improve diabetes and its complications.

Among Firmicutes, *F. prausnitzii* is the most abundant species in human and plays an important role in the healthy gut [99]. The consumption of a higher quantity of animal meat, animal fat, sugar, processed foods, and low fiber diet (the typical westernized diet) reduces the count of *F. prausnitzii*, while a high-fiber (vegetables and fruits) and low meat diet enhance the count of *F. Prausnitzii* [99]. It is known to consume a variety of diet containing polysaccharides, such as the prebiotic inulin, arabinoxylans, apple pectin, oligofructose, resistant starch, fructan supplement, pectins and some host-derived carbon sources (including d-glucosamine and *N*-Acetyl-d-glucosamine) [99,197]. Polysaccharides generally serve as the primary modulators of the function and composition of gut microbiota. They are mostly consumed in the food due to their relative safety, availability and low cost. Increased consumption of polysaccharides is likely to be of benefit to individuals, who follow a typical Western-style diet, most of whom consume adequate amounts of dietary fibre [198]. Meta-analyses also show that the increased consumption of fibre significantly reduces the risk of mortality [199,200]. The study stated that the consumption of low-fat, high-complex carbohydrate diet (LFHc) increases the abundance of *F. prausnitzii* and protective effects against diabetes (evaluated with the Oral Glucose Tolerance Test), as suggested by the findings of an improvement in insulin sensitivity [199]. The composition of the LFHc diet was 28% fat (12% monounsaturated; 8% polyunsaturated and 8% saturated) and Mediterranean diet was 35% fat (22% monounsaturated; 6% polyunsaturated and 7% saturated). These data suggest that the long-term consumption of the LFHc and Mediterranean diet could be a therapeutic and preventive tool for diabetes, and increase the abundance of *F. prausnitzii* [200]. An earlier study, *F. prausnitzii* has been proposed as potent probiotics for the treatment of gut inflammation [201]. Hence, the modulations and abundance of *F. prausnitzii* are occurring through the consumption of prebiotics and/or probiotics and/or formulations. Long-term consumption of these compositions may help prophylactic or therapeutic applications for metabolic diseases, including diabetes. This could open a new hypothesis to be tested in the future in bigger populations about whether the consumption of healthy diets reduces the risk of diabetes by influencing the *F. prausnitzii* profile (Figure 5).

## 6. *F. prausnitzii* Transplantation Improves Diabetes

*F. prausnitzii* is one of the most common and abundant gut microbiota belonging to the *Clostridium leptum* cluster IV, promotedt by a plant-based carbohydrate-rich diet [202]. It is unique and active commensal intestine bacterium and the representative of phylum—*Firmicutes*, class—*Clostridium* and family—*Ruminococcaceae* placed over 5% of the total gut microbiota population in the healthy human gut [203]. *F. prausnitzii* is a butyrate-producer and has well known the anti-inflammatory potential in the host [46]. Normally, butyrate gives energy for the host (5–15% of the total calories) that protects against pathogenic invasion, modulates the immune system and inhibits cancer progression [203], as well as autoimmune diabetes [204]. Moderate butyrate levels can also prevent high-fat-diet-induced insulin insensitivity through epigenetic regulation, and mitochondrial beta-oxidation [205]. *F. prausnitzii* is one of the unique organisms that reduce various autoimmune diseases, especially type-1 diabetes via the modulation of gut epithelium homeostasis and immune system [206]. Studies associated with gut microbiota and type-1 diabetes in animals and human subjects showed an alteration with a lower proportion of butyrate-producing organisms, such as *Firmicutes* and *Clostridium*, which protects against autoimmune diabetes [14,162,207]. *F. prausnitzii* might also regulate the development of autoimmune diabetes via butyrate dependent complementary pathways [208,209]. An abundant quantity of butyrate can even lower the gut barrier function and enhance cell apoptosis [158]. High levels of butyrate stimulate GLP-1 secretion and enhance insulin sensitivity through cAMP signal, such as PKA and Epac, which inhibits gastric emptying in humans [3]. Due to the inhibition of gastric emptying, butyrate can be excreted slowly and accumulates, influencing the anti-inflammatory potential, pH, and oxidative stress.

It has been well known that changes in the abundance of *F. prausnitzii* have been associated with dysbiosis with various illnesses in human [210]. The count of *F. prausnitzii* significantly decreased in diabetic individuals with negative correlation to glycated hemoglobin HbA1c values [158,159]. However, this abundancy is connected with the decreased level of NF-κB, IL-8 and the elevated levels of IL-12, IFN-γ, and IL-10, that often linked with cancers [154], type-2 diabetes [211], inflammatory bowel disease [187], Crohn’s disease [212], and Colitis [201]. Xu et al. [213] reported that a Chinese herbal formula alleviated fasting blood glucose and HbA1c levels that were associated with an abundance of *F. prausnitzii*. Along with *Akkermansia muciniphila*, *F. prausnitzii* abundantly found in individuals with normal glucose tolerance compared to the pre-diabetic subjects [161]. However, the higher abundance of *F. prausnitzii* is controversially found in obese Indian children when compared with non-obese controls [214]. *F. prausnitzii* has been suggested as a marker for a healthy gut. It can convert acetate into butyrate using butyryl-CoA: Acetate CoA-transferase (BUT) pathways and thereby providing the balanced pH in the gut [158]. *F. prausnitzii* contributes an adequate butyrate production based on BUT gene in lean controls (15%) when compared with the obese (40%) and diabetes group (42%) [158]. High-fat diets supplemented with butyrate prevented insulin resistance in obese mice [195,215]. Remely et al. [46] also reported a lower proportion of inflammatory markers found with *F. prausnitzii* in diabetic subjects, indicating a higher incidence of low-grade inflammation. An elevated level of butyrate is considered to inhibit the diet-induced obesity [216] and cause suppression of inflammatory reactions [199]. Altogether, butyrate alone did not provoke the observed inhibitory effect, demonstrating that *F. prausnitzii* likely secretes an unknown anti-inflammatory metabolite apart from the butyrate [197].

*F. prausnitzii* transplantation is an effective therapeutic approach for diabetes and its complications. Vrieze et al. [217] investigated the effects of infusing the gut microbiota from lean donors to male recipients with metabolic syndrome. In this study, the team substantiated the human colonization with *F. prausnitzii* used as a probiotic; further they found that the phyla *Firmicutes* quantitatively 2–3 fold increased after allogenic infusion. Small intestinal biopsy results also showed that *E. coli* increased 2.21-fold with autologous infusion and decreased 0.58-fold with allogeneic infusion; fecal *Ruminococcus bromii* increased 1.65-fold with autologous infusion and increased 2.49-fold with allogeneic infusion. Finally, the team had suggested that butyrate producing bacteria prevent translocation of endotoxic compounds derived from the gut microbiota, which has been demonstrated to drive insulin resistance. Similarly, another study also suggested that the butyrate synthesizing microbiota could improve insulin sensitivity through signaling pathways and direct effect on glucose metabolism [218].

Sokol et al. [201] found the transplantation of *F. prausnitzii* in mice protects the gut epithelium and inhibit experimentally induced gut inflammation. In addition, an in vitro study also suggested the human immune cells with *F. prausnitzii* exhibit a potential anti-inflammatory response in the gut [201]. Hence, *F. prausnitzii* transplantation prevents gut altered microbiota causing low-grade inflammation and protects the pancreas from autoimmunity. Transplantation of intestinal microbiota especially *F. prausnitzii* from a normal individual to metabolic syndrome subjects, especially diabetic persons, is able to synthesize abundant quantity of butyrate, which stabilizes the leaky gut and inhibits downstream pro-inflammatory mechanisms. An earlier study of fecal microbiota transplantation has demonstrated to heal recurrent infection with *Clostridium difficile* and directly addressed whether the gut microbiota can affect the host metabolism [219]. Infusion of donor feces was significantly more effective for the treatment of recurrent *C. difficile* infection than the use of antibiotics. Similarly, this study would help to establish the rapid detection of *F. prausnitzii* abundance and warrants further investigation as a biomarker of intestinal health and metabolic disorders. To improve the understanding of how the microbiota affects the metabolism in humans, metagenomics, transcriptomics, proteomics and metabolomics data from key target tissues and the microbiota during various disease states and interventions should be combined to provide a map of co-occurrences. These data enable the formation of testable hypotheses that can be pursued in validated animal models, and they will form the foundation for precise interventions.

## 7. Conclusions

The diet provides not only energy to the host, but also modulates and maintains the symbiotic gut microbiota. Intake of a complex diet and fibers enables to enhance the production of SCFAs and helps to maintain various microbiota compositions and impacting host-microbe interactions. SCFAs production is normally associated with the greater number of *Bacteroides* and *F. prausnitzii*, which are the consistent manufacturer of propionate and butyrate respectively. Both compositions are potent health-promoting effects and protect from chronic inflammation in the gut and thereby prevent metabolic disorders, including diabetes. The consumption of high-fat plus high-carbohydrate meal induces endotoxemia and inflammation in the gut. However, the consumption of high-fruit plus high fiber meal or vegetarian diet modulate microbial ecology, reduces low-grade inflammations and are effective therapeutic treatments for many diet-associated metabolic diseases. Information of the role gut microbiota (*F. prausnitzii*) plays in diabetes could be used to advance intervention strategies to avert and/or treat disparities that prime to treat the inflammation preceding overt manifestations of metabolic disorders. The transplantation of *F. prausnitzii* is an effective therapeutic approach for diabetes and its complications [217]. It has also been proposed *F. prausnitzii* as potent probiotics and consumption of these compositions may help prophylactic or therapeutic applications for diabetes. However, well-controlled prospective human studies are quite mandatory to advance an understanding of the influence of *F. prausnitzii* and its functions to environmental factors. Such information could be used to categorize effective preventive strategies targeting precise factor of the gut ecosystem.

## Figures and Tables

**Table 1:** Association between the diet and the gut microbiota.

Diet Components/Sources	Consumption of Dietary Sources	Changes in the Gut Bacteria
Carbohydrates: Indigestible complex oligosaccharides	Human milk glycans [69,70,71]	↑*Bifidobacterium infantis*, *Bacteroides*
	Resistant starch (type 2,3,4) [72,73]	*RS2:* ↑*Ruminococcus bromii*, ↑*Eubacterium rectale* *RS3:* ↑*Ruminococcus bromii*, ↑*Ocillobactor*, ↑*Eubacterium rectale* *RS4:* ↑*Bifidobacterium adoloscentis*, ↑*Parabacteroides distasonis*
	Resistant maltodextrin [74]	↑*Ruminococcus*, ↑*Eubacterium*, ↑*Lachnospiraceae*, ↑*Bacteriodes*, ↑*Holdemania*, ↑*Faecalibacterium*
	Jerusalem artichoke inulin [75,76,77]	↑*Bifidobacterium*, ↑*Lactobacillus*, ↑*Enterococcus*, ↑*Faecalibacterium prausnitzii*, ↑*Clostridial cluster XIVa*
	Inulin and partially hydrolysed guar gum, fructo-oligosaccharides, Long chain inulin, Xylo- oligosaccharides [78,79,80,81]	↑*Lactobacillus*/*Enterococcus,* ↑*Bifidobacterium*, ↓*Clostridium*, ↓*Bacteroides*, ↓*Prevotell*
	Galacto-oligosaccharides, fructo-oligosaccharides [82,83]	↑*Bifidobacterium*
	Polydextrose and soluble corn fibre [84,85]	↑*Clostridiaceae*, ↑*Faecalibacterium prausnitzii*, ↑*Phasolarciobacterium*, ↑*Dialister*, ↑*Lactobacillus*, ↑*Ruminococcus intestinalis,* ↓*Eubacteiaceae*, *Actinobacteria*
Simple sugars Digestible carbohydrates	Arabinoxylans [86,87]	↑*Bifidobacteria*, ↑*Eubacteriumrectale*, ↑*Roseburia*/*Eubacterium*, ↑*Faecalibacterium prausnitzii*, ↑*Bacteroides*
	Sugars in food [61]	↑*Prevotella*
Insoluble complex carbohydrates	Apple [88]	↑*Bifidobacteria*, ↓*Clostridia perfringens*
	Kiwifruit [89]	↑*Bifidobacteria*, ↑*Lactobacillus*, ↑*Clostridia*
	Banana [90]	↑*Bifidobacterium*
Insoluble non-starch polysaccharides	Cereal cellulose [91,92,93,94]	↑*Ruminococcus flavefaciens*, ↑*Clostridium xylanolyticum*
	Cereal amylose [91,92,93,94]	↑*Clostridium butyricum*
	Amylopectin and Starch [91,92,93,94,95,96,97,98]	↑*Clostridium ramosum*, ↑*Clostridium cluster XIVa*, ↑*Bacteroides*
		↑*Ruminococcus bromii*, ↑*Eubacterium rectale,* ↑*Roseburia*
		↑*E. rectale*, ↑*Roseburia*
	Dietary fiber [99]	↑*Clostridium*, ↑*Bacteroides*, ↑*Bacillus subtilis*, ↑*Bifidobacterium*,↑*Fusobacterium*
	Soybean, radishes, cabbage, fish, seaweed and green tea [41] Western diet (high in meat) [41]	↓*Bacteroides fragilis;* ↑*Lactobacillus*, ↑*E. coli*, ↑*Proteus*, ↑*Klebsiella*, ↑*Staphylococcus*, ↑*Streptococci*, ↑*Clostridium*, ↑*Eubacterium*, ↑*Ruminococcus*
	Cereal (millet, grain, sorghum), Legumes (black-eyed peas) and Vegetables [100]	↑*Bacteroidetes*, ↑*Prevotella,* ↑*Xylanibacter*, ↓*Firmicutes*
	Whole grain wheat [101,102]	↑*Bifidobacteria animalis*, ↑*Roseburia,* ↑*Bacteroides*, ↑*Prevotella*, ↑*Clostridium* ↑*Lactobacillus/Enterococci*
	Maize-based whole grains and cereals [103]	↑*Bifidobacterium spp.,* ↑*Atobobium cluster spp.*
	Whole grain barley, brown rice or mix [104]	↑*Firmicutes*, ↑*Blautia*, ↑*Roseburia*, ↑*Bifidobacterium*, ↓*Bacteroides*, ↓*odoribacter*
	Rye bread [105]	↓*Bryantella formatexiagans*
	Wild blueberry drink [106,107]	↑*Bifidobacterium spp.,* ↑*Lactobacillus acidophilus*, ↑*B. longum sub sp. infantis*
	Red wine, dealcoholized red wine, gin [108]	↑*Bacteroidetes*, ↑*Bacteroides*, ↑*B. uniformis*, ↑*Firmicutes*, ↑*E. rectale group*, ↑*Prevotella*, ↑*Fusobacteria*, ↑*Proteobacteria*, ↑*Bifidobacterium*, ↑*Eggerthellalenta*, ↑*Enterococcus*
	Almonds and pistachios [109,110]	↑*Bifidobacterium*, ↑*Lactobacillus spp.,* ↓*Lactic acid bacteria*, ↓*Clostridumperfringens*
Fat and fatty acids	High-fat diet [111,112]	↑*E. rectal*, ↑*C. coccoides*, ↓*Bifidobacterium*, ↓*Bacteroides*,
Protein	Meat [113,114]	↑*Bacteroides*, ↑*Bifidobacterium*, ↑*Peptococcus*, ↑*Lactobacillus,* ↑*Clostridium cluster XIVa,* ↑*Clostridium coccoides,* ↑*Roseburia*, ↑*E. rectal*
	A variety of amino acids and saturated fats [61,100]	↑*Bacteroides*, ↑*Clostridium*
	Whey protein isolate [115]	↑*Lactobacillaceae*, ↓*Clostridiaceae*
	Chickpea or raffinose [116]	↓*Clostridium cluster I*, *II XI*
	Soymilk, low glycinin soymilk, bovine milk [117]	↑*Bacteroides*, ↑*prevotella*, ↑*Lactobacillus*, ↓*Bifidobacterium*
Non-nutrients (Phytochemicals)	Red pepper (*Capsicum annuum*) and Garlic (*Allium sativum*) [118,119]	↓*Bacillus cereus*, ↑*B. subtilis*, ↑*C. tetani*, ↑*Helicobacter pylori*
	Tea polyphenols [120]	↓*Bacteroides*, ↓*Clostridium perfringens,* ↓*C. difficile*, ↓*E. coli,* ↓*Salmonella typhimurium*
	Wild blueberries (*Vaccinium angustifolium*) [106,121]	↑*Bifidobacterium,* ↑*Lactobacillus acidophilus,* ↑*Bacteroides*, ↑*Prevotella*, ↑*Enterococcus*, ↑*C. coccoides*
	Coffee (catechin and epicatechin) [122]	↑*C. coccoides*, ↑*E. rectale group,* ↑*E. coli*, ↓*C. histolyticum*
	Dietary polyphenol [123]	↑*Bifidobacterium*, ↑*Lactobacillus*
	Wine (resveratrol) [124,125]	↑*Bifidobacterium*, ↑*Lactobacillus,* ↓*Proteus mirabilis*
	Berries (anthocyanins) [126,127]	↓*Staphylococcus*, ↓*Salmonella*, ↓*H. pylori*, ↓*B. cereus*

↑ increase; ↓ decrease.

**Table 2:** Alteration of bacterial species associated with type 2 diabetes.

Name of the Prevalence Bacteria	Model	References
↑*Akkermansia muciniphila*, ↑*Bacteroides intestinalis*, ↑*Bacteroides sp.* ↑*Clostridium bolteae*, ↑*Clostridium ramosum*, ↑*Clostridium sp. HGF2*, ↑*Clostridium symbiosum*, ↑*Colstridium hathewayi*, ↑*Desulfovibrio sp.*, ↑*Eggerthellalenta*, ↑*Escherichia coli*	Human	[156]
↑*Bacteroides*, ↑*Prevotella*, ↑*Clostridia*, ↑*Betaproteobacteria*, ↑*Bacteroidetes*/*Firmicutes* ratio, ↓*Firmicutes*, ↓*Clostridia*, ↓*Eubacterium rectale*	Human	[157]
↑ *Bacteroidetes thetaiotaomicron*, ↑*Akkermansia muciniphila*,↑*E. coli*	Human	[142,158]
↓*Faecalibacterium prausnitzii phylotypes*	Human	[159]
↑*Betaproteobacteria*, ↓*Firmicutes (Clostridia)*	Mice	[27]
↓*Bifidobacterium*, ↓*Bacteroides vulgatus*	Human	[61]
↓*Bacteroidescaccae*, ↓*Eubacteriumrectale*, ↓*Faecalibacterium prausnitzii*, ↓*Roseburia intestinalis*, ↓*Roseburiainulinivorans*, ↑*Clostridium hathewayi*, ↑*Clostridium ramosum*, ↑*Clostridium symbiosum*, ↑*Eggerthellalenta*, ↑*Escherichia coli*, ↑*Akkermansia muciniphila*, ↑*Desulfovibrio*, ↑*Clostridiales sp. SS3/4*,	Mice	[160]
↑*Lactobacillus spp.*, ↑*Clostridium clostridioforme*, ↓*Roseburia*, ↓*Clostridium spp.*	Human	[161]
↑*Akkermansia muciniphila*, *↑Faecalibacterium prausnitzii*, ↓*Bacteroides*	Human	[162]
↑*Bifidobacterium*, ↑*Bacteroides*, ↑*Lactobacillus*, ↑*Lactococcus*, ↑*Streptococcus*, ↑*Veillonella*, ↑*Alistipes*, ↓*Prevotella*, ↓*Akkermansia*, ↓*Eubacterium*, ↓*Fusobacterium*, ↓*Anaerostipes*, ↓*Roseburia*, ↓*Subdoligranulum*, ↓*Faecalibacterium*	Human	[13]
↑*Bacteroides*, ↑*Bacteroidesovatus*, ↑*Eubacterium*, ↓*Faecalibacterium*, ↓*Bacteroides vulgatus*, ↓*Bacteroidesfragilis*	Human	[163]
↑*Bacteroides*, ↑*Veillonella*, ↑*Clostridium*, ↑*Prevotella*, ↓*Lactobacillus*, ↓*Bifidobacterium*, ↓*Blautiacoccoides*, ↓*Eubacteriumrectale*	Human	[164]
↑*Cantidaalbicans*, ↑*Enterobacteriaceae*, ↑*Echerichia coli,* ↓*Bifidobacterium*	Human	[165]

↑ increase; ↓ decrease.

**Table 3:** Diagnostic and therapeutic implications of *F. prausnitzii* on various gut-associated disorders.

Gut-Associated Diseases	Findings	Implications	References
**Diagnostic implications of** ***F. prausnitzii***			
Inflammatory bowel diseases	↑ *F. prausnitzii* counts in feces	*F. prausnitzii* assay might play a potentially useful adjunct role in non-invasive screening and diagnosis of inflammatory bowel diseases	[181]
Inflammatory bowel diseases associated with skin disorders	↓ *F. prausnitzii* and ↑ *E. coli*	*F. prausnitzii* assay aids to identify IBD-associated skin disorders	[182,183]
Crohn’s disease	↑ *F. prausnitzii* counts with acidic stool	*F. prausnitzii* assay gives a promising diagnostic biomarker for early Crohn’s disease	[184]
Crohn’s disease	↑ bilirubin concentrations along with *F. prausnitzii* counts with acidic stool	*F. prausnitzii* analysis contributes a promising diagnostic biomarker for Crohn’s disease	[185]
Colorectal cancer	↓ *F. prausnitzii* counts in feces	*F. prausnitzii* assay holds great promising as a diagnostic biomarker for early colon cancer detection and monitoring and has considerable potential for developing an anticancer therapy	[186]
Ulcerative colitis	↓ *F. prausnitzii* counts in feces	*F. prausnitzii* analysis contributes a promising diagnostic biomarker for Ulcerative colitis	[186]
Irritable bowel syndrome	↓ *F. prausnitzii* counts in feces	*F. prausnitzii* phylotypes quantified as a putative biomarker and depicting the significance of the loss of these subtypes in Irritable bowel syndrome pathogenesis.	[187]
Crohn’s disease, ulcerative colitis, and colorectal cancer	↓ *F. prausnitzii* phylogroup I was found in subjects with Crohn’s disease, ulcerative colitis, and colorectal cancer, whereas phylogroup II was specifically reduced in with Crohn’s disease.	Quantification of *F. prausnitzii* phylogroups and *E. coli* may help to identify gut disorders and to classify inflammatory bowel disease location.	[188]
**Therapeutic implications of *F. prausnitzii* in gut-associated diseases**			
Gut-associated diseases	Treatment with *F. prausnitzii* as probiotics can inhibit gut-associated diseases, including malignancy	*F. prausnitzii* as next-generation probiotics might be useful in the treatment of various cancers with gut-associated diseases	[189]
Low-grade inflammation	Treatment with *F. prausnitzii* as probiotics exhibited intestinal permeability, tissue cytokines, and serotonin levels	*F. prausnitzii* might be beneficial effects on intestinal epithelial barrier impairment in a chronic low-grade inflammation model.	[190]
Inflammatory bowel diseases	Treatment with *F. prausnitzii* as probiotics showed plasma anti-Th17 cytokines (IL-10 and IL-12) and reduced IL-17 levels in both plasma and colonic mucosa, with ameliorated colonic colitis lesions	*F. prausnitzii* protected the colon mucosa against the development of Inflammatory bowel diseases and suggesting a promising therapy for Inflammatory bowel diseases.	[191]
Crohn’s disease	Seven peptides were identified in the *F. prausnitzii* culture, known as anti-inflammatory molecules. These molecules reduce the activation of the NF-kB pathway with a dose-dependent effect in the dinitrobenzene sulfonic acid induced-colitis model	*F. prausnitzii* protected the colon mucosa against the development of Inflammation and suggesting a promising treatment for Crohn’s disease	[192]
Ileal Crohn’s disease	Oral administration of *F. prausnitzii* as probiotics showed as anti-inflammatory properties. They reduce IL-1beta-induced NF-κB pathway in dinitrobenzene sulfonic acid induced-colitis model	*F. prausnitzii* as a probiotic is a promising strategy in Crohn’s disease	[193,194]
Ulcerative Colitis	Oral administration of *F. prausnitzii* reduced Th1, Th2, and Th17 immune response and increased TGFβ production.	*F. prausnitzii* as a probiotic is a promising strategy in Colitis	[195]
Crohn’s disease	Oral administration of *F. prausnitzii* as probiotics showed as anti-inflammatory properties. They induced IL-10, an anti-inflammatory cytokine, in peripheral blood mononuclear cells	*F. prausnitzii* strains could represent good candidates as next-generation probiotic.	[196]

↑ increase; ↓ decrease.

## Abbreviation

AP1	Activator protein 1
ATP	Adenosine monophosphate
BUT	Butyryl-CoA:acetate CoA-transferase
cAMP	Cyclic adenosine monophosphate
CD14	Cluster of differentiation 14
CREB	Cyclic adenosine monophosphate responsive element-binding protein
DPP-IV	Dipeptidyl peptidase IV
Epac1 and Epac2	Exchange protein directly activated by cyclic adenosine monophosphate 1 and 2
ES	Embryonic stem cells
EX 4	Exendin 4
GI tract	Gastrointestinal tract
GIP	Gastric inhibitory peptide
GLP-1	Glucagon-like peptide-1
GPCR	G-protein coupled receptors
GPR41 and GPR43	G-protein receptors 41 and 43
IEC	Intestinal epithelial cell
IFN-γ	Interferon gamma
IL	Interleukin
IRAKs	Interleukin 1receptor associated kinases
IRF	Interferon regulatory factors
LBP	Lipopolysaccharide binding protein
LFHc	Low-fat, high-complex carbohydrate diet
LPS	Lipopolysaccharide
MAL	Myeloid differentiation primary response protein 88-adaptor-like protein
MAPK	Mitogen-activated protein kinases
MLDS	multiple low-dose streptozotocin
MYD88	Myeloid differentiation primary response protein 88
NF-κB	Nuclear factor kappa B
PCNA	Proliferating cell nuclear antigen
PDX-1	Pancreatic and duodenal homeobox 1
PKA	Protein kinase A
PYY	Peptide YY
SCFA	Short chain fatty acid
SUR1	Sulfonylurea receptor 1
TGFβ	Tumour growth factor beta
TLR4	Toll-like receptor 4
TNF	Tumour necrosis factor
TRAF	Tumour necrosis factor receptor-associated factors
Tregs	Regulatory T cells
